# Conversion of Sewage Water into H_2_ Gas Fuel Using Hexagonal Nanosheets of the Polyaniline-Assisted Deposition of PbI_2_ as a Nanocomposite Photocathode with the Theoretical Qualitative Ab-Initio Calculation of the H_2_O Splitting

**DOI:** 10.3390/polym14112148

**Published:** 2022-05-25

**Authors:** N. M. A. Hadia, Mohammed A. H. Khalafalla, Fatma M. Abdel Salam, Ashour M. Ahmed, Mohamed Shaban, Aljawhara H. Almuqrin, Ali Hajjiah, H. A. Hanafi, Mansoor Alruqi, Abdel-Hamid I. Mourad, Mohamed Rabia

**Affiliations:** 1 Physics Department, College of Science, Jouf University, Sakaka P.O. Box 2014, Al-Jouf, Saudi Arabia; nmhadia@ju.edu.sa; 2 Basic Sciences Research Unit, Jouf University, Sakaka P.O. Box 2014, Al-Jouf, Saudi Arabia; 3 Department of Physics, Faculty of Science, Taibah University, Yanbu P.O. Box 41315, Saudi Arabia; mkhalfalla@taibahu.edu.sa; 4 Nanophotonics and Applications Lab, Physics Department, Faculty of Science, Beni-Suef University, Beni-Suef 62514, Egypt; fatmamohamed922@yahoo.com (F.M.A.S.); ashour.mohamed@science.bsu.edu.eg (A.M.A.); mssfadel@aucegypt.edu (M.S.); mohamedchem@science.bsu.edu.eg (M.R.); 5 Nanomaterials Science Research Laboratory, Chemistry Department, Faculty of Science, Beni-Suef University, Beni-Suef 62514, Egypt; 6 Physics Department, Faculty of Science, Islamic University of Madinah, Madinah 42351, Saudi Arabia; 7 Department of Physics, College of Science, Princess Nourah bint Abdulrahman University, Riyadh 11671, Saudi Arabia; ahalmoqren@pnu.edu.sa; 8 Electrical Engineering Department, College of Engineering and Petroleum, Kuwait University, Safat 13113, Kuwait; 9 Chemistry Department, College of Science and Humanities Al-Quwayiyah, Shaqra University, Al-Quwayiyah 19257, Saudi Arabia; hanafia020@gmail.com; 10 Cylotron Project, Nuclear Research Center, Egyptian Atomic Energy Authority, Cairo P.O. Box 13759, Egypt; 11 Department of Mechanical Engineering, College of Engineering, Shaqra University, Riyadh 11911, Saudi Arabia; malruqi@su.edu.sa; 12 Mechanical and Aerospace Engineering Department, College of Engineering, United Arab Emirate University, Al Ain 15551, United Arab Emirates; 13 Mechanical Design Department, Faculty of Engineering, Helwan University, Cairo 11795, Egypt

**Keywords:** polyaniline, PbI_2_, hydrogen generation, nanocomposite, water splitting, sewage water

## Abstract

This study is very promising for providing a renewable enrgy (H_2_ gas fuel) under the elctrochemical splitting of the wastwater (sewage water). This study has double benefits: hydrogen generation and contaminations removel. This study is carried out on sewage water, third stage treated, from Beni-Suef city, Egypt. Antimony tin oxide (ATO)/polyaniline (PANI)/PbI_2_ photoelectrode is prepared through the in situ oxidative polymerization of PANI on ATO, then PANI is used as an assistant for PbI_2_ deposition using the ionic adsorption deposition method. The chemical structural, morphological, electrical, and optical properties of the composite are confirmed using different analytical tools such as X-ray diffreaction (XRD), scanning electron microscope (SEM), transmision electron microscope (TEM), Fourier-transform infrared spectroscopy (FTIR), and UV-Vis spectroscopy. The prepared PbI_2_ inside the composite has a crystal size of 33 nm (according to the peak at 12.8°) through the XRD analyses device. SEM and TEM confirm the hexagonal PbI_2_ sheets embedded on the PANI nanopores surface. Moreover, the bandgap values are enhanced very much after the composite formation, in which the bandgap values for PANI and PANI/PbI_2_ are 3 and 2.51 eV, respectively. The application of ATO/PANI/PbI_2_ nanocomposite electrode for sewage splitting and H_2_ generation is carried out through a three-electrode cell. The measurements carreid out using the electrocehical worksattion under th Xenon lamp (100 mW.cm^−2^). The produced current density (J_ph_) is 0.095 mA.cm^−2^ at 100 mW.cm^−2^ light illumination. The photoelectrode has high reproducibility and stability, in which and the number of H_2_ moles is 6 µmole.h^−1^.cm^−1^. The photoelectrode response to different monochromatic light, in which the produced J_ph_ decreases from 0.077 to 0.072 mA.cm^−2^ with decreasing of the wavelengths from 390 to 636 nm, respectively. These values confirms the high response of the ATO/PANI/PbI_2_ nanocomposite electrode for the light illuminaton and hydrogen genration under broad light region. The thermodynamic parameters: activation energy (Ea), enthalpy (ΔH*), and entropy (ΔS*) values are 7.33 kJ/mol, −4.7 kJ/mol, and 203.3 J/mol.K, respectively. The small values of ΔS* relted to the high sesnivity of the prepared elctrode for the water splitting and then the hydrogen gneration. Finally, a theoretical study was mentioned for calculation geometry, electrochemical, and thermochemistry properties of the polyaniline/PbI_2_ nanocomposite as compared with that for the polyaniline.

## 1. Introduction

The huge demand for energy sources all over the world drives scientists to concentrate all efforts on renewable energy sources [1,2,3,4]. These renewable energy sources provide replaceable energy sources for people to overcome the problem related to fossil fuels. These fuels have limited energy sources with additional fetal hazardous effects on the livings and the environment. There are famous hazardous gases are confirmed such as SO_X_, CO_X_, NO_X_ usually release from these fossil fuels [5,6,7].

One of the most important renewable energy sources is solar energy. Through this solar energy, the scientists worked on the photocatalytic materials that can use this energy for performing additional chemical reactions through which the H_2_ gas is evolved. This H_2_ gas is very important as a fuel for factories and companies, in addition to its use as a fuel for the usual working inside the home such as warming and cooking. 

This H_2_ gas resulted from the water-splitting reaction by receiving electrons from the photocatalytic semiconductor nanomaterials such as metal oxides and nitrides [8,9]. The enhancements in these materials are carried out by increasing the active sites in their surface area, this is carried out through the preparation of these materials in nanoscale with great surface morphologies such as nanowire, nanotube, and nanosheets [10,11,12].

One of the great challenges is the application of polymer nanomaterials as semiconductor photocatalytic for the replacement of the previous semiconductor materials. The polymer materials have great properties such as large surface area, mass production, and low cost. PANI and its derivatives are considered semiconductor materials with high electrical and optical properties qualified them for photocatalytic applications. Moreover, this category of the polymer has additional properties such as high safety, stability, compatibility, redox state, and low bandgap [13,14].

Recently, few studies have concentrated on using PANI or conductive polymers as a photocatalytic material for water-splitting reactions [15,16,17,18,19,20,21]. Belabed et al. [21] prepared PANI/TiO_2_ as catalytic material for water splitting under artificial light. Zhang et al. [22] studied PANI/MoS_2_ for H_2_ generation by using H_2_SO_4_ as sacrificing agent, in which the J_ph_ value was 0.09 mA.cm^−2^. Corte et al. [20] fabricated Ni/PANI composite for water-splitting reaction by H_2_SO_4_ as an electrolyte, in which the J_ph_ value was 0.091 mA.cm^−2^. In addition to that, there were studies carried out on poly(3-aminobenzoic acid) frame as photocatalyst for H_2_ generation through using H_2_SO as an electrolyte, the J_ph_ was 0.08 mA.cm^−2^ [15]. In addition to that, the applications of metal oxides and nitrides have a great advantage of high stability. These metals are deposited by high complexed techniques with high costs such as physical vapor deposition, RF sputtering, and laser techniques [16,17].

There are many drawbacks to the previous studies related to the water-splitting reaction. This literature depended on using sacrificing agents for water spitting reactions such as Na_2_SO_3_, Na_2_S_2_O_3_, HCl, and NaOH [18,19,20]. These sacrificing agents have a great role in electrode corrosion and then decreasing the lifetime of the electrode. Moreover, the previous studies have very small J_ph_ values released from the splitting reaction, this confirmed that these studies have very small efficiency for the splitting reaction [21,22,23,24]. In addition to that, these previous studies usually used freshwater as a source of H_2_, this was a big problem due to the leakage in freshwater that is used as drinking water. 

In this study, wastewater is used as a source for renewable energy production (H_2_ gas). PANI/PbI_2_ composite is prepared using a very cheap method, in which PANI is used as an assistant for the deposition of PbI_2_ through the ionic adsorption deposition method on the supporter ATO glass. The ATO/PANI/PbI_2_ nanocomposite is used as a working electrode for the wastewater-splitting reaction, in which sewage water (the third stage treated) is used as an electrolyte without using any additional sacrificing agent. 

This study provides H_2_ gas from wastewater, at the same time, this study decreases using of fossil fuels with their harmful fetal effects related to their hazardous gases. 

Here, ATO/PANI/PbI_2_ nanocomposite is used as a working electrode in a three-electrode cell, in which graphite and saturated calomel represent the counter and references electrodes. The effects of light wavelengths on/off chopped light and reproducibility are studied. The number of H_2_ moles is calculated through Faraday’s laws. The thermodynamic parameters are calculated using Eyring equations. Moreover, the simple mechanism for the water-splitting reaction is mentioned. 

Soon, we will work on synthesis an industrial model of an electrochemical cell for the industrial applications. This electrochemical cell can be used inside the home for converting the sewage water into H_2_ gas directly that can be used as fuel for warming and cooking directly. Moreover, this model can be used inside the economic companies and factories with high financial returns.

## 2. Materials and Methods

### 2.1. Preparation of PANI/PbI_2_ Nanocomposite

The preparation of PANI occurred under the in situ oxidation polymerization of aniline on the antimony tin oxide (ATO) glass (Sigma Aldrich, St. Louis, MO, USA). A total of 50 mL (0.1 M) of aniline (El Nasr co., Cairo, Egypt) was dissolved under the ultrasonic for 30 min in the presence of (0.5 M) CH_3_COOH (El Nasr co., Cairo, Egypt) as acid medium and solvent.

In a parallel flask, 50 mL (0.15 M) of (NH_4_)_2_S_2_O_8_ (Piochem co., Cairo, Egypt) was dissolved well that represents the oxidant. (NH_4_)_2_S_2_O_8_ is added suddenly over the aniline solution, through this process the polymerization of aniline to PANI took place and led to the formation of green color indicated the formation of PANI. Finally, ATO/PANI thin film was washed with distillated water and dried at 60 °C for 6 h.

The deposition of PbI_2_ over the ATO/PANI thin film occurred using the ionic adsorption precipitation method. ATO/PANI was immersed in (0.05 M) Pb(NO_3_)_2_ (Piochem co., Cairo, Egypt) solution for 2 h at 298 K. through this process, the adsorption of Pb^2+^ ions occur and led to the formation of ATO/PANI/Pb^2+^. This thin film was dried well and immersed in (0.01 M) iodine solution at 25 °C for 15 min. The reaction between I^−^ and Pb^2+^ was completed and led to the deposition of PbI_2_ on the ATO/PANI thin film and then the formation of ATO/PANI/PbI_2_ thin film.

**Figure 1:**
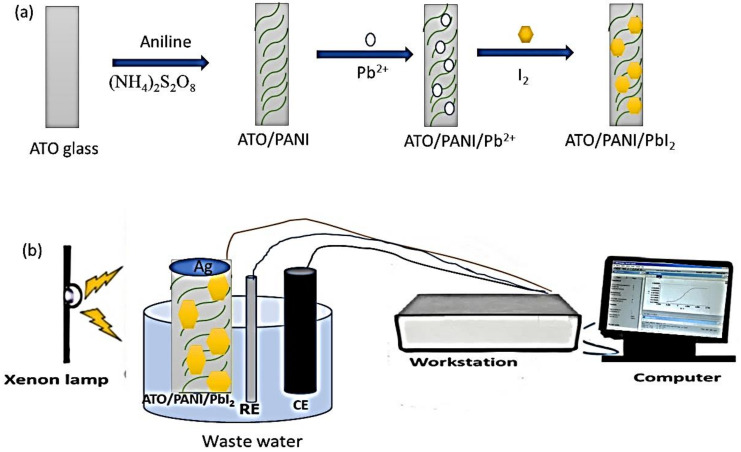
The schematic diagram for the preparation of ATO/PANI/PbI_2_ nanocomposite electrode., aniline polymerization using the oxidant (NH)_2_S_2_O_8_, then the PbI_2_ deposition in two steps (**a**) and (**b**) the electrochemical measurements using the three-electrode cell under Xenon lamp.

From this reaction, the deposition of PbI_2_ occurred on the surface and inside the polymer chains, as shown in the schematic diagram (Figure 1a).

### 2.2. Materials

(NH_4_)_2_S_2_O_8_ and aniline were obtained from Winlab (UK) and Rankem (India) companies, respectively. CH_3_COOH, Iodine (I_2_), Pb(NO_3_)_2_, and KI were purchased from ElNaser company (Egypt). The wastewater, i.e., sewage water, was obtained from the drinking water sanitation company, in which this wastewater was treated three stages (the third stage treatment), this company located in Beni-Suef city, Egypt.

### 2.3. Characterization and Analyses

X-ray diffractometer (PANalytical Pro, Holland, Almelo, The Netherlands) was used for chemical structure determination and crystal size calculation. Fourier transform infrared was used for confirming the chemical structure, FTIR 340 Jasco spectrophotometer (Easton, WA, USA). A scanning electron microscope was used for determining the morphology of the prepared samples (SEM) (ZEISS, Gemini, Column, Oberkochen, Germany). In the same manner, a transmitted electron microscope was used for determining the internal morphology of the samples (TEM) (JEOL JEM-2100, Oberkochen, Germany). The optical absorption and then the bandgap calculation was determined through the Shimadzu UV/Vis spectrophotometer, Waltham, MA, USA. ImageJ software was used for the calculation of the surface morphology and cross-section. 

### 2.4. The Electrochemical Test

The electrochemical measurements were carried out using a power station (CHI660E), Austin, USA, under a Xenon lamp, Waltham, USA, as shown in Figure 1b. The measurements were carried out through a three-electrodes cell. ATO/PANI/PbI_2_, graphite, and satureated calomel were used as working, counter, and references electrodes, respectively. Sewage water was used as an electrolyte without using any additional sacrificing agents.

### 2.5. The Theoretical Calculation

All the calculations were performed using Orca software [25] with def2-SVP (Karlsruhe basis def2-SVP Split valence polarization [26] basis sets and def2/J auxiliary sets for all atoms except Pb and I for which def2-TZVP basis was assigned during the structural geometry optimizations. The calculation employs the atom-pairwise dispersion correction with the Becke–Johnson damping scheme (D3BJ [26]. We used the conductor-like polarizable continuum model (CPCM) [27] solvation model properties to emulate the water medium for the reaction. The default self-consistent (SCF) setting for the energy calculations was employed. To further accelerate the geometry and frequency (for infra-red (IR) calculations) convergence, we adopted the “sloppy SCF” settings.

## 3. Results and Discussion

### 3.1. Characterization of the Prepared Nanomaterials

**Figure 2:**
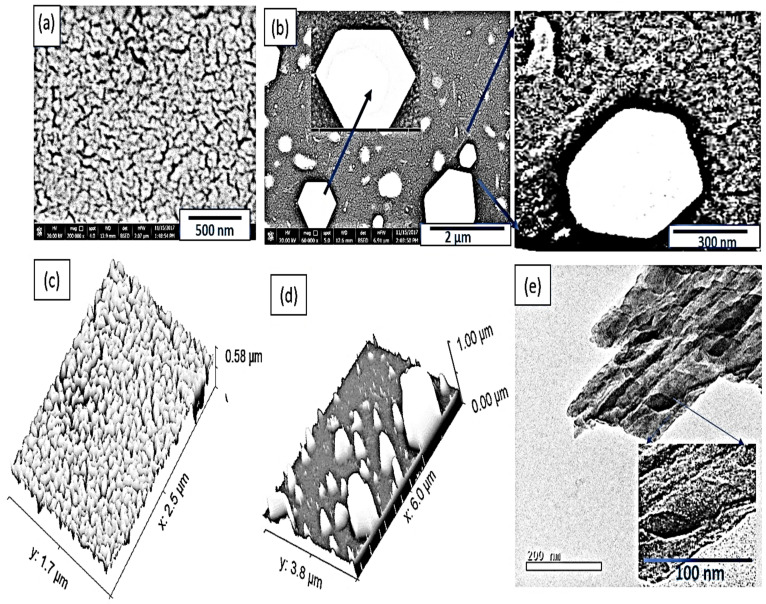
The SEM and modeling images for (**a**,**c**) PANI, (**b**,**d**) PANI/PbI_2_ nanomaterials. (**e**) TEM image of the PANI/PbI_2_ nanocomposite.

The morphology of the prepared PANI is shown in Figure 2a, moreover the software Image J program modeling [28] is shown in Figure 2c. From both figures, the formation of a homogeneous nonporous surface appears clearly. The great roughness and small porousness in the PANI surface qualify it for composite well with additional nanomaterials. 

The morphology of the prepared PANI/PbI_2_ is shown in Figure 2b. The hexagonal PbI_2_ sheets cover the PNAI network, at the same time, these particles are embedded through the polymer network and cover its fibers. This feature was confirmed well through ImageJ software, as shown in Figure 2. The thickness of the film is greater in comparison with the PANI surface. The PbI_2_ in the composite appears as an obelisk over the PANI surface. The average diameter of the PbI_2_ sheets is about 300 nm. 

The TEM image of the composite PANI/PbI_2_ is shown in Figure 2e. The hexagonal PbI_2_ shape appears well (dark color) through the PANI surface. For more confirmation, other SEM figures is inserted under different scale bars as shown in Figure S1a,b.

The great contact and homogenous morphology of the prepared composite qualify this composite for photocatalytic reactions very well. The composite will have the optical properties combined with its two materials.

**Figure 3:**
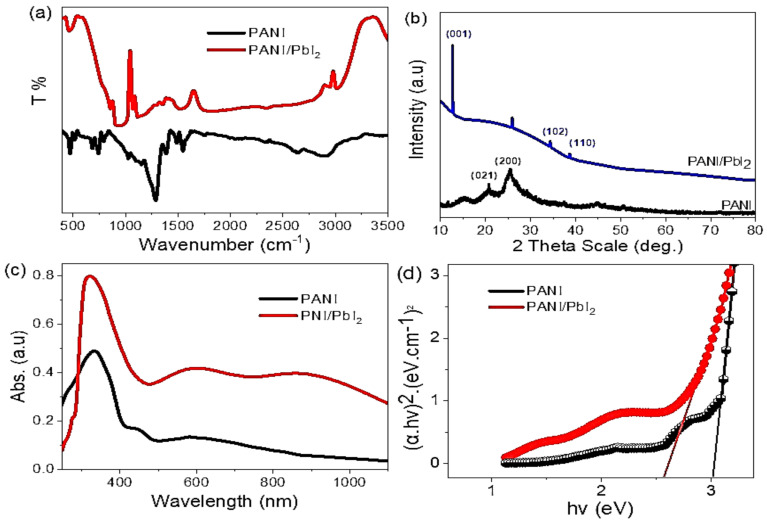
(**a**) XRD, (**b**) FTIR, (**c**) optical absorbance, (**d**) bandgap of PANI and PANI/PbI_2_ nanomaterials.

The chemical structures of the prepared PANI and PANI/PbI_2_ nanomaterials are confirmed using the FTIR as shown in Figure 3a. The summarized data is mentioned in Table 1. From this Figure and Table, the functional groups are mentioned that are related to the polymer and PbI_2_ nanomaterials. The heteropolar diatomic molecule PbI_2_ vibration stretching appears at 1370 and 1010 cm^−1^. The function groups related to the PANI are located at 3401, 2918, 1105 cm^−1^, for N–H, C–H, C–N aromatic, respectively. The function group C=C for the quinoid and benzene rings are located at 1467 and 1301 cm^−1^, respectively. While the para disubstituted is located at 587 cm^−1^. There are some shifts in the function groups after the composite formation, in which N–H, C=C quinoid, C–N are shifted to 3424, 1470, 1105 cm^−1^, respectively. While there is a blue shift in the C=C benzenoid ring to 1291 cm^−1^.

The XRD pattern of PANI and PANI/PbI_2_ nanomaterials is shown in Figure 3b. For PANI, the XRD sharp peak at 20.7° and semi-sharp peak at 25.5° indicate the crystalline nature of PANI nanomaterial. These two peaks are located at the growth directions of (021) and (200), respectively.

After the PANI/PbI_2_ composite, there is a shift in the peak at 25.5° to 26.05° with the appearance of a new peak at 20.7°. Moreover, there is the appearance of three peaks at 12.8, 34.4, and 38.7° corresponding to the growth directions of (001), (102), and (112), respectively. The standard XRD pattern is mentioned in Figure S2. These characteristic peaks are related to the PbI_2_ nanomaterials inside the composite. The crystal size of the prepared nanomaterial is calculated using Scherrer’s formula, Equation (1) [31,32]. This equation depends on many factors such as the width half maximum (W), the X-ray wavelength (λ), dimensionless factor (k), and Bragg angle (θ). From this Equation, the crystal size of the PbI_2_ inside the composite is 33 nm according to the peak at 12.8°.

D = 0.9λ/W cosθ(1)

(2) $$\alpha h\nu \;=\;A{\left(h\nu -E_{g}\right)}^{1/2}$$

(3) $$\alpha =\left(\frac{2303}{d}\right)A$$

The optical analyses of the prepared PANI and PANI/PbI_2_ nanocomposite are shown in Figure 3c. From this Figure, there is more enhancement in the optical behavior after the composite formation. This is related to the enhancement in the optical absorption intensity and the position of the peaks. For both the PANI and PANI/PbI_2_, there is a peak in the UV region at 325 nm, but the intensity of this peak increases very much after the composite formation. This peak is related to the band-to-band electron transition process. 

Moreover, there is a redshift from 585 nm to 610 nm after the composite formation, with the appearance of a great peak at 877 nm in the IR region. This peak is related to the electron vibration process. 

Thus, there are more enhancements in the optical properties of the composite compared to the PANI material. This is related to the hexagonal sheets of PbI_2_ that absorb and capture the photons. Then, they used these photons for hot electron generation through the formation of a hole electron bandgap. 

The bandgap values of the PANI and PANI/PbI_2_ are calculated from Tauc’s equations, Equations (2) and (3) as shown in Figure 3. These equations depend on absorption coefficient (α), absorbance (A), frequency (ν), and Planck constant (h). The bandgap values are enhanced very much after the composite formation, in which the bandgap values for PANI and PANI/PbI_2_ are 3 and 2.51 eV, respectively. 

This enhancement in the optical properties of the composite matched well with the good crystalline structure as shown by the XRD analysis before. So, the prepared PANI/PbI_2_ composite is qualified for photocatalytic applications and water-splitting reactions.

### 3.2. Photoelectrochemical Water-Splitting Reaction 

The electrochemical measurements of the wastewater (sewage water, third treated) splitting were carried out using the PowerStation (CHI660E) under a Xenon lamp. The ATO/PANI/PbI_2_ nanocomposite represents the working electrode, while graphite and saturated calomel represent the counter and reference electrodes, respectively. 

**Figure 4:**
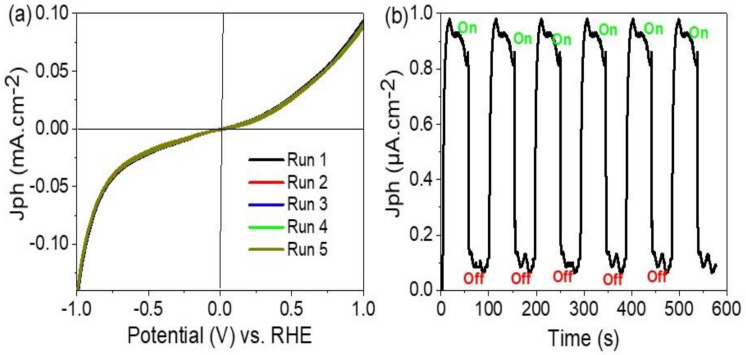
(**a**) The J_ph_—potential relation with repeating five runs, (**b**) on/off chopped light for the prepared ATO/PANI/PbI_2_ nanocomposite photoelectrode at 25 °C.

The electrochemical splitting reaction was carried out through the sewage water without using any additional sacrificing agent, the chemical composition of the sewage water is mentioned in Table 2. The measurements were carried out at 25 °C with a sweep rate of 100 mV.s^−1^. Under the photon incidence, there is charge transfer due to the splitting in the PANI levels, in which there is electron transfer from the LUMO to HOMO. The energy level of HOMO is higher than the conducting band of the PbI_2_, so there is energy transfer and collection of electrons over the conducting band of PbI_2_. Although there is a Schottky barrier [33] that affects the electron transfer from the PANI to PbI_2_ and causes slow motion of electrons that appear in the behavior of the J_ph_-potential relation (Figure 4a). This depletion layer does not affect the electron transfer, in which the produced J_ph_ value is 0.095 mA.cm^−2^ at 100 mW.cm^−2^, and finally, the electrons reach the water molecules for the spitting process and H_2_ generation reaction.

The relation between the applied potential (−1 to +1V) and the produced J_ph_ values for the prepared electrode is shown in Figure 4a. This relation is repeated five times under the same conditions, the J_ph_ value is 0.095 mA.cm^−2^ with high reproducibility. The standard deviation is very small (at about 1%). 

Under a very small bias voltage, the on/off chopped current is shown in Figure 4b. The J_ph_ values change from 0.1 to 0.98 µA.cm^−2^, these values indicate the response of the prepared electrode for light sensitivity. This high sensitivity is related to the role of PbI_2_ that captures and traps the photons, in which these photons generate hot electrons that do oscillations and resonance on the PbI_2_ surface. Moreover, the generated electrons on PANI combine with these electrons for creating a high flow of electrons that transfer to the neighbor H_2_O molecules for water-splitting reaction, and then H_2_ and O_2_ evolution reaction.

The chopped current is repeated with high reproducibility, this indicates the high stability of the electrode for a long time [34,35,36]. This is related to the high stability of PANI chains, in which PANI is not dissolved in almost all the solvents [37]. The very small dark current (J_d_) (almost zero) may indicate the full inhibition of the prepared electrode under dark conditions [38,39,40]. 

The number of H_2_ moles is calculated from the Faraday law relation [5]. This law depends on the parameters; J_ph_, time change (dt), the molecular weight of H_2_ gas, oxidation number (z), and Faraday constant (F, 9.65 × 10^4^ C mol^−1^). Through this relation, 6 µmole.h^−1^.cm^−1^ of H_2_ gas evolved as small bubbles from the cell using the prepared photoelectrode.

(4) $$H_{2}(\mathrm{moles})=\int_{0}^{t}\frac{J_{\mathrm{ph}}\mathrm{dt}}{F}\cdot M/z$$

**Figure 5:**
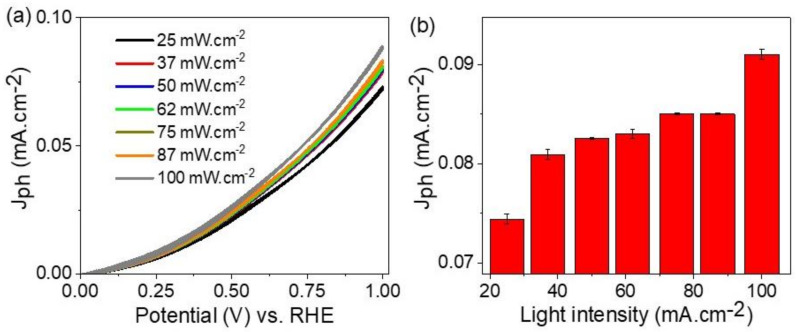
(**a,b**) The response of ATO/PANI/PbI_2_ photoelectrode for the various light intensities, using J_ph_—potential relation.

The effects of light intensities from 25 to 100 mW.cm^−2^ on the ATO/PANI/PbI_2_ appear clearly through the produced J_ph_ values as shown in Figure 5a. The J_ph_ values increase from 0.075 to 0.092 mA.cm^−2^ with increasing in the light intensities from 25 to 100 mW.cm^−2^, respectively. This behavior is confirmed well in Figure 5b, in which the produced J_ph_ values (at 1.0 V) are shown under various light intensities. 

The increases in J_ph_ values are related to increasing of photons numbers (N) through increasing the light intensities (P) [7]. This relation is confirmed through Equation (5) using different parameters: wavelength (λ), light velocity (c), and Planck constant (h).

The photon flux is received through the photocatalytic material surface that activates the active sites [41]. Through this process, the splitting in the outer energy levels takes place and led to the production of hot electrons [42,43,44,45,46]. These electrons are collected on the surface of the photocatalytic material and cause the production of J_ph_ [47]. Thus, with increasing of the hot electrons, the J_ph_ value increase represents the rate of water-splitting reaction, and hence the rate of H_2_ evolution.

(5) N=λP/hc

**Figure 6:**
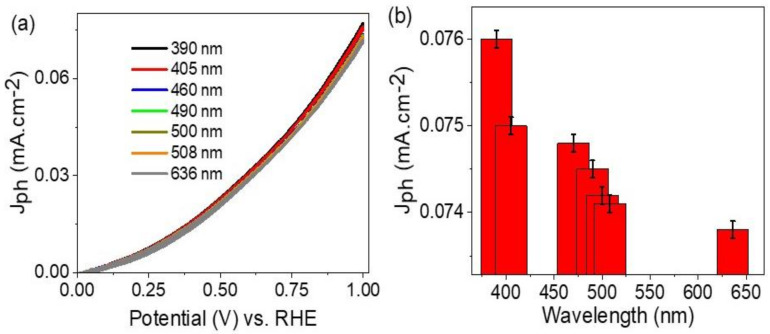
(**a**) The response of the ATO/PANI/PbI_2_ photocathode for various light wavelengths. (**b**) The produced J_ph_ values at 1.0 V and 25 °C.

The effect of monochromatic wavelengths on the ATO/PANI/PbI_2_ photoelectrode is shown in Figure 6a. The prepared photoelectrode responds well to the various wavelengths, in which the produced J_ph_ values decrease from 0.077 to 0.073 mA.cm^−2^ with increasing of the light wavelengths from 390 to 636 nm, respectively. The produced J_ph_ values at 1.0 V under different monochromatic light are shown in Figure 6b. The decreasing of the J_ph_ values with increasing of the wavelengths matches well with the optical absorption curve for the composite as mentioned before in Figure 3c. The high J_ph_ values in the blue side are related to the high light frequency with high energy that causes electrons to transfer to the conducting band that appears as J_ph_ values [37]. 

**Figure 7:**
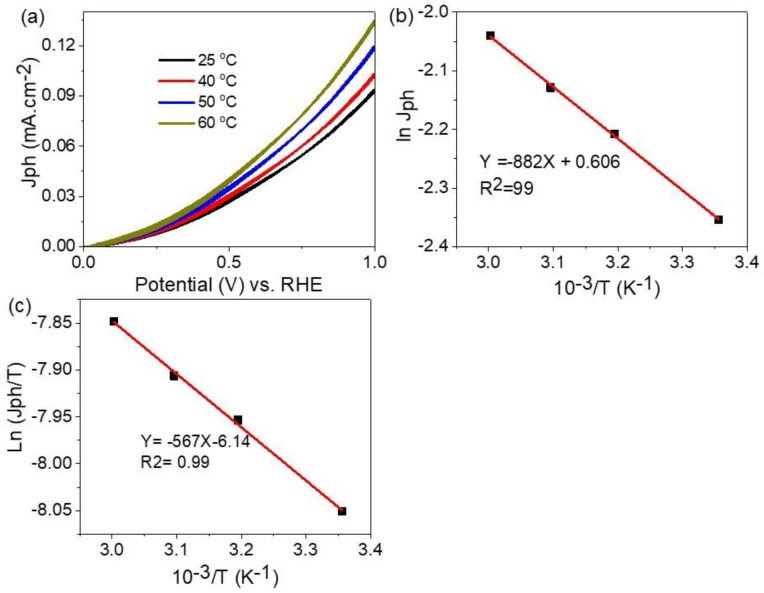
(**a**) The effect of temperature on ATO/PANI/PbI_2_ photoelectrode. (**b,c**) The relation between Ln J_ph_ and Ln J_ph_/T with the reciprocal of temperature, respectively, in the temperature range from 25 to 60 °C.

The response of the prepared ATO/PANI/PbI_2_ photoelectrode for different temperatures (25 to 60 °C) is shown in Figure 7a. From this figure, the produced J_ph_ values increase from 0.092 to 0.132 mA.cm^−2^ with increasing in the temperature from 25 to 60 °C, respectively. The high values of J_ph_ at high temperature relate to the mobility of the increasing ions with temperature. This behavior indicates the increase of the water splitting, and hence the rate of H_2_ generation with the increasing of the temperature [48].

The thermodynamic parameters (Ea, ΔS, and ΔH) are calculated through the Eyring equations: Equation (6) with Equation (7) [5,49,50] and using Figure 7b,c. The calculation is based on the constants: k_B_, h, k and R which are Boltzmann’s, Planck’s, reaction rate, and universal gas constants, respectively. From Equation (6) and Figure 7b, the Ea is 7.33 kJ/mol. While from Equation (7) and Figure 7c, the ΔH* and ΔS* values are −4.7 kJ/mol and 203.3 J/mol.K, respectively. The positive ΔS*, negative ΔH*, and small Ea values indicate the spontaneous H_2_ generation reaction [51].

(6) $$k={\mathrm{Ae}}^{-\mathrm{Ea}/\mathrm{RT}}$$

(7) $$k=T\cdot \frac{\mathrm{kB}}{h}\cdot {\;e}^{\Delta S/R}\cdot e^{-\Delta H/\mathrm{RT}}$$

The comparison of the electrolyte used and the produced J_ph_ of the prepared ATO/PANOI/PbI_2_ photoelectrode with the previous literature is mentioned in Table 3. From this comparison, although the previous literature usually uses sacrificing agents, their produced J_ph_ values are still very small. The present study uses sewage water only, the produced J_ph_ value is higher than the previous work. In addition to that, the prepared electrode has high advantages represented in the low cost, easily prepared, high stability, and reproducibility. These great properties qualify the prepared ATO/PANOI/PbI_2_ photoelectrode for industrial applications. 

### 3.3. The Theoretical Study

**Figure 8:**
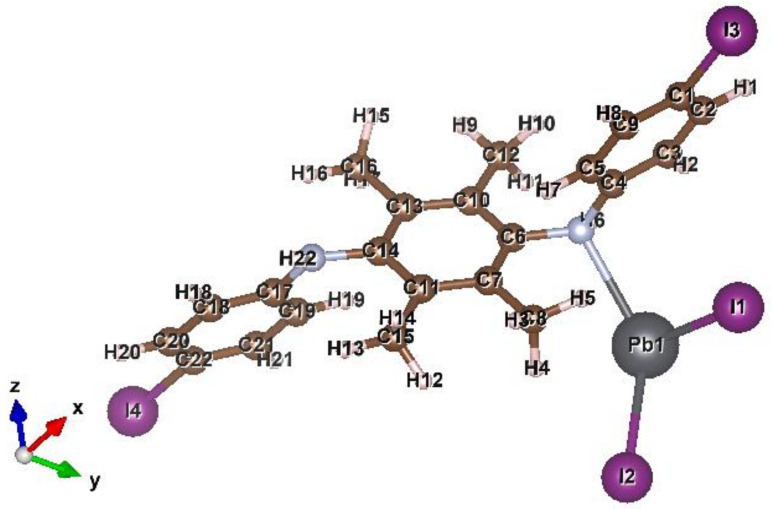
Optimized geometry of the PANI/PbI_2_ composite. The binding energy between PbI_2_ and the polyaniline.

The geometrically optimized PANI/PbI_2_ composite structure is shown in Figure 8. The computational procedure of the H_2_O splitting on the Pb site of the composite structure involves fixing the composite’s atoms while freeing the H_2_O molecule.

Table 4 shows the calculated electrochemical and thermochemistry properties of the PANI/PbI_2_ nanocomposite as compared with that for the PANI. Interesting, an increase in the molecular energy gap between the frontier orbitals (HOMO and LUMO for highest occupied molecular orbital and lowest unoccupied molecular orbital, respectively) is observed due to the composite formation. The gap might be exaggerated due to the default high Hartree Fock portion used in the Hamiltonian during the SCF calculation. Nevertheless, our calculated effects of the composite formation on the electrochemical properties are expected to convey the essential information for comparison with the experiments. Table 4 also shows that electronegativity (χ), global hardness (η), and electrophilicity (ω) are enhanced due to composite formation. The negative difference between the Gibbs free energies for the composite and polymer indicates the spontaneous formation of the composite. Additionally, we have calculated the binding energy between PbI_2_ and the polyaniline at −0.05 Ha, suggesting a stable composite structure. The composite exhibits a significant increase in the dipole moment as compared to the polymer. This may effectively influence the catalytic effect of the composite for splitting H_2_O into H_2_.

We point out that the infrared (IR) calculations for the composite revealed few imaginary frequency modes that could have been ruled by using more stringent calculation settings such as hybrid functionals and larger basis sets [56]. However, these are computationally expensive and may not affect our current qualitative conclusions. 

**Figure 9:**
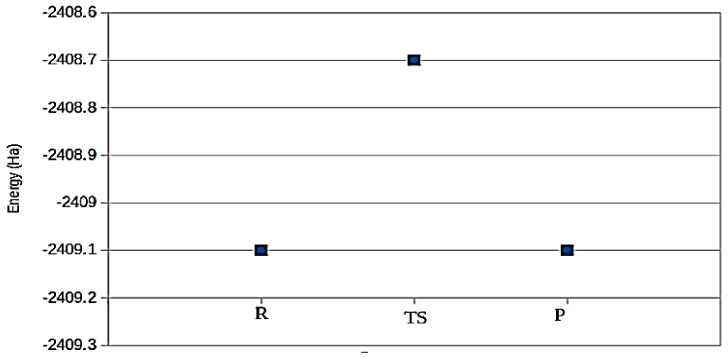
The main energies along the reaction line are from the reactant state (R), through the tran− sition state (TS), to the product state (P). The structures corresponding to these states are shown in Figure 10.

**Figure 10:**
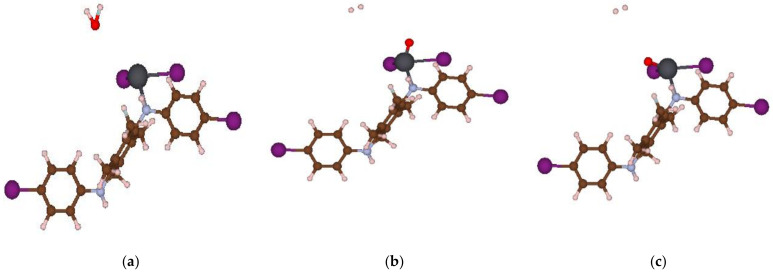
Atomic configurations of the reactant (R)—(**a**) transition state (TS)—(**b**) and product (P)—(**c**) along the reaction line for the H_2_O splitting by the PbI_2_-Polyaniline.

Figure 9 shows the energetics for the reaction path with reactants (R) formed by a free H_2_O molecule and the composite, and a product (P) representing a free H_2_ and a non-bonded structure between O and the composite. The transition state structure (TS) is shown with higher energy than that for the P and R. Investigation of the IR frequency modes indicates that the TS structure is less stable compared to the P structure. The structures along the reaction path are indicated in Figure 10. Again, since we focused on the qualitative results, we did not employ the systematic procedure of the Nudged Elastic Band (NEB) for finding the TS state [57]. Moreover, we have qualitatively demonstrated the possible reaction towards water splitting into H_2_ by using the bare composite. A more realistic and systematic (catalytic-like) reaction may be achieved via supporting the composite on a suitable metallic layer. We will postpone this to future investigations. 

## 4. Conclusions

ATO/PANI/PbI_2_ nanocomposite photoelectrode was prepared and used for H_2_ generation from sewage water. The sewage water was related to Beni-Suef city, Egypt. 

The preparation of PANI was carried out through in situ polymerization on the ATO electrode. This film was used as an assistant for the deposition of PbI_2_ through the ionic adsorption deposition method. From the characterization devices, the crystal size of the composite was 33 nm, with a bandgap of 2.46 eV. The PbI_2_ has hexagonal sheets embedded in the PANI nanopores surface. 

The ATO/PANI/PbI_2_ photoelectrode was applied for H_2_ generation from sewage water through a three-electrodes cell. The rate of H_2_ generated is estimated through J_ph_ values. The J_ph_ was 0.095 mA.cm^−2^ at 100 mW.cm^−2^. The response of the electrode to various wavelengths was carried out, in which the J_ph_ values decreased from 0.077 to 0.073 mA.cm^−2^ with decreasing of the wavelengths from 390 to 636 nm, respectively. The on/off chopped current confirmed the high sensitivity and reproducibility of the prepared photoelectrode. The thermodynamic parameters were calculated and confirmed the high efficiency of the electrode for H_2_ generation reaction, in which Ea, ΔH*, ΔS* values were 7.33 kJ/mol, are −4.7 kJ/mol, and 203.3 J/mol.K, respectively. Finally, a theoretical study was mentioned for showing the geometry of the nanocomposite and calculation of some parameters such as dipole moment, HOMO, and LUMO energy for the PANI/PbI_2_ composite as compared to the PANI. Soon, our team working on synthesis an industrial model of elctrochemical cell that can convert the sewage water into hydrogn gas direclty. This idea is very promising for providing hydrigen gas fuel for people in houses for warming and cooking. Moreover, providing hydrogen fuel for people in remote places or inside the deserts.

## Figures and Tables

**Table 1:** The FTIR spectra of PANI and PANI/PbI_2_ nanocomposite.

Band Position (cm^−1^)		Assignment
PANI/PbI_2_	PANI	
3424	3401	N–H group [29]
2923 2856	2918	C–H aromatic ring group [29]
1470	1467	C=C of the quinoid ring [30]
1375	-	heteropolar diatomic molecules
1291	1301	C=C of benzenoid rings [30]
1108	1105	C–N
1010	1049	Heteropolar diatomic molecules of PbI_2_
792	789	C–H in-plane [29]
590	587	Para disubstituted aromatic rings

**Table 2:** The chemical composition of the sewage water (third treated stage) electrolyte used for H_2_ generation reaction.

Material or Element	Concentration (mg/L)
Phenols	0.015
F^−^	1.0
Al^3+^	3.0
NH_3_	5.0
Hg^2+^	0.005
Pb^2+^	0.5
Cd3^+^	0.05
As^3+^	0.05
Cr^3+^	1.0
Cu^2+^	1.5
Ni^3+^	0.1
Fe^3+^	1.5
Mn^2+^	1.0
Zn^2+^	5.0
Ag^+^	0.1
Ba^3+^	2.0
Co^2+^	2.0
Other cations	0.1
Pesticides	0.2
CN^−1^	0.1
Industrial washing	0.5
Coli groups	4000/100 cm^3^

**Table 3:** The electrolyte used and J_ph_ value of the present work in comparison with the previous literature.

Photoelectrode	Electrolyte	J_ph_ (mA/cm^2^)
Ni/PANI [20]	H_2_SO_4_	0.091
PANI/MoS_2_ [22]	H_2_SO_4_	0.09
Poly(3-aminobenzoic acid) frame [15]	H_2_SO_4_	0.08
g–C_3_N_4_–CuO [52]	NaOH	0.01
CuO–C/TiO_2_ [53]	glycerol	0.012
TiN–TiO_2_ [54]	NaOH	3.0 × 10^−4^
BiFeO_3_ [21]	NaOH	0.09
Au/Pb(Zr, Ti)O_3_ [55]	NaOH	0.06
ATO/PMT/PbI_2_ (present work)	Sewage water	0.095

**Table 4:** Some of the calculated electrochemical and thermal properties of the optimized PI_2_-Poly structure.

	PbI_2_-Polyaniline	Polyaniline
E_HOMO_, eV	−7.9119	−7.8566
E_LUMO_, eV	0.6318	3.1765
Electronegativity (χ), eV	3.64005	2.34005
Global hardness (η), eV	4.27185	5.51655
Electrophilicity (ω), eV	1.5509	0.49631
Total dipole moment, Debye	12.56297	0.00019
Gibbs free energy (G), Ha	−2332.91	−1547.50

## Data Availability

The data that support the findings of this study are available from the corresponding author upon reasonable request.

## Supplementary Materials

The following are available online at https://www.mdpi.com/article/10.3390/polym14112148/s1, Figure S1. The SEM of PANI/PbI_2_ under different scale bars (**a**) 5 μm and (**b**) 1 μm; Figure S2. The standard XRD for PANI/PbI_2_.

## References

[1] Nishiyama H., Yamada T., Nakabayashi M., Maehara Y., Yamaguchi M., Kuromiya Y., Nagatsuma Y., Tokudome H., Akiyama S., Watanabe T. (2021). Photocatalytic solar hydrogen production from water on a 100-m^2^ scale. Nature.

[2] Hisatomi T., Domen K. (2019). Reaction systems for solar hydrogen production via water splitting with particulate semiconductor. Nat. Catal..

[3] Pagliaro M. (2019). Preparing for the future: Solar energy and bioeconomy in the United Arab Emirates. Energy Sci. Eng..

[4] Takata T. (2020). Photocatalytic water splitting with quantum efficiency of almost unity. Nature.

[5] Mohamed F., Rabia M., Shaban M. (2020). Synthesis and characterization of biogenic iron oxides of different nanomorphologies from pomegranate peels for efficient solar hydrogen production. J. Mater. Res. Technol..

[6] Shaban M., Ali S., Rabia M. (2019). Design and application of nanoporous graphene oxide film for CO_2_, H_2_, and C_2_H_2_ gases sensing. J. Mater. Res. Technol..

[7] Elsayed A.M., Rabia M., Shaban M., Aly A.H., Ahmed A.M. (2021). Preparation of hexagonal nanoporous Al_2_O_3_/TiO_2_/TiN as a novel photodetector with high efficiency. Sci. Rep..

[8] Kang Z., Cheng Y., Zheng Z., Cheng F., Chen Z., Li L., Tan X., Xiong L., Zhai T., Gao Y. (2019). MoS_2_-Based Photodetectors Powered by Asymmetric Contact Structure with Large Work Function Difference. Nano-Micro Lett..

[9] Lee J.H., Lee W.W., Yang D.W., Chang W.J., Kwon S.S., Park W. (2018). Il Anomalous Photovoltaic Response of Graphene-on-GaN Schottky Photodiodes. ACS Appl. Mater. Interfaces.

[10] Nourisabet T., Jamshidi Aval H., Shidpour R., Naji L. (2022). Fabrication of a PEO-PVDF blend based polymer composite electrolyte with extremely high ionic conductivity via the addition of LLTO nanowires. Solid State Ion..

[11] Sarkar J., Ganguly S. (2022). Investigation of the thermal properties of Cu–Ag core-shell nanowires using molecular dynamics simulation. Phys. B Condens. Matter.

[12] Wang Y., Yang Z., Wu Q., Liu W., Li Y., Zhang H., Ma X., Cong L., Wang H., Zhang D. (2022). Effect of stacking faults on magnetic properties and magnetization reversal in Co nanowires. Mater. Charact..

[13] Abukhadra M.R., Rabia M., Shaban M., Verpoort F. (2018). Heulandite/polyaniline hybrid composite for efficient removal of acidic dye from water; kinetic, equilibrium studies and statistical optimization. Adv. Powder Technol..

[14] Shaban M., Abukhadra M.R., Rabia M., Elkader Y.A., Abd El-Halim M.R. (2018). Investigation the adsorption properties of graphene oxide and polyaniline nano/micro structures for efficient removal of toxic Cr(VI) contaminants from aqueous solutions; kinetic and equilibrium studies. Rend. Lincei.

[15] Modibane K.D., Waleng N.J., Ramohlola K.E., Maponya T.C., Monama G.R., Makgopa K., Hato M.J. (2020). Poly(3-aminobenzoic acid) Decorated with Cobalt Zeolitic Benzimidazolate Framework for Electrochemical Production of Clean Hydrogen. Polymers.

[16] Chiang C.Y., Aroh K., Franson N., Satsangi V.R., Dass S., Ehrman S. (2011). Copper oxide nanoparticle made by flame spray pyrolysis for photoelectrochemical water splitting—Part II. Photoelectrochemical study. Int. J. Hydrogen Energy.

[17] Guo X., Diao P., Xu D., Huang S., Yang Y., Jin T., Wu Q., Xiang M., Zhang M. (2014). CuO/Pd composite photocathodes for photoelectrochemical hydrogen evolution reaction. Int. J. Hydrogen Energy.

[18] Teixeira G.F., Silva Junior E., Vilela R., Zaghete M.A., Colmati F. (2019). Perovskite Structure Associated with Precious Metals: Influence on Heterogenous Catalytic Process. Catalysts.

[19] Mishra M., Chun D.M. (2015). α-Fe_2_O_3_ as a photocatalytic material: A review. Appl. Catal. A Gen..

[20] Acar C., Dincer I., Naterer G.F. (2016). Review of photocatalytic water-splitting methods for sustainable hydrogen production. Int. J. Energy Res..

[21] Liu G., Karuturi S.K., Chen H., Wang D., Ager J.W., Simonov A.N., Tricoli A. (2020). Enhancement of the photoelectrochemical water splitting by perovskite BiFeO_3_ via interfacial engineering. Sol. Energy.

[22] Wang Z., Cao D., Wen L., Xu R., Obergfell M., Mi Y., Zhan Z., Nasori N., Demsar J., Lei Y. (2016). Manipulation of charge transfer and transport in plasmonic-ferroelectric hybrids for photoelectrochemical applications. Nat. Commun..

[23] Freeman E., Kumar S., Thomas S.R., Pickering H., Fermin D.J., Eslava S. (2020). PrFeO_3_ Photocathodes Prepared Through Spray Pyrolysis. ChemElectroChem.

[24] Sherman B.D., Ashford D.L., Lapides A.M., Sheridan M.V., Wee K.R., Meyer T.J. (2015). Light-Driven Water Splitting with a Molecular Electroassembly-Based Core/Shell Photoanode. J. Phys. Chem. Lett..

[25] Neese F., Wiley J. (2012). The ORCA program system. Wiley Interdiscip. Rev. Comput. Mol. Sci..

[26] Huzinaga S., Andzelm J. (1984). Gaussian Basis Sets for Molecular Calculations.

[27] Cossi M., Rega N., Scalmani G., Barone V. (2003). Energies, structures, and electronic properties of molecules in solution with the C-PCM solvation model. J. Comput. Chem..

[28] Saad R., Gamal A., Zayed M., Ahmed A.M., Shaban M., BinSabt M., Rabia M., Hamdy H. (2021). Fabrication of ZnO/CNTs for Application in CO_2_ Sensor at Room Temperature. Nanomaterials.

[29] Sayyah S.M., Azooz R.E. (2016). Electrosynthesis and characterization of adherent poly(2-aminobenzothiazole) on Pt-electrode from acidic solution. Arab. J. Chem..

[30] Sayyah S.M., Shaban M., Rabia M. (2016). A High-Sensitivity Potentiometric Mercuric Ion Sensor Based on m-Toluidine Films. IEEE Sens. J..

[31] Basak M., Rahman M.L., Ahmed M.F., Biswas B., Sharmin N. (2022). The use of X-ray diffraction peak profile analysis to determine the structural parameters of cobalt ferrite nanoparticles using Debye-Scherrer, Williamson-Hall, Halder-Wagner and Size-strain plot: Different precipitating agent approach. J. Alloy. Compd..

[32] Burton A.W., Ong K., Rea T., Chan I.Y. (2009). On the estimation of average crystallite size of zeolites from the Scherrer equation: A critical evaluation of its application to zeolites with one-dimensional pore systems. Microporous Mesoporous Mater..

[33] Almohammedi A., Shaban M., Mostafa H., Rabia M. (2021). Nanoporous TiN/TiO_2_/Alumina Membrane for Photoelectrochemical Hydrogen Production from Sewage Water. Nanomaterials.

[34] Yang S., Huang S., Jiang Q., Yu C., Zhou X. (2022). Experimental study of hydrogen generation from in-situ heavy oil gasification. Fuel.

[35] Aazam E.S., Zaheer Z. (2022). Silver-Cobalt bimetallic nanoparticles to the generation of hydrogen from formic acid decomposition. Arab. J. Chem..

[36] Xiao F., Yang R., Liu Z. (2022). Active aluminum composites and their hydrogen generation via hydrolysis reaction: A review. Int. J. Hydrogen Energy.

[37] Abdelazeez A.A.A., El-Fatah G.A., Shaban M., Ahmed A.M., Rabia M. (2021). ITO/Poly-3-Methylaniline/Au Electrode for Electrochemical Water Splitting and Dye Removal. ECS J. Solid State Sci. Technol..

[38] Kenfoud H., Nasrallah N., Baaloudj O., Belabed C., Chaabane T., Trari M. (2022). Opto-electrochemical characteristics of synthesized BaFe_2_O_4_ nanocomposites: Photocatalytic degradation and hydrogen generation investigation. Int. J. Hydrogen Energy.

[39] Liu Z., Xiao F., Tang W., Cong K., Li J., Yang R., Hao J. (2022). Study on the hydrogen generation performance and hydrolyzates of active aluminum composites. Int. J. Hydrogen Energy.

[40] Keshipour S., Asghari A. (2022). A review on hydrogen generation by phthalocyanines. Int. J. Hydrogen Energy.

[41] Elsayed A.M., Shaban M., Aly A.H., Ahmed A.M., Rabia M. (2022). Preparation and characterization of a high-efficiency photoelectric detector composed of hexagonal Al_2_O_3_/TiO_2_/TiN/Au nanoporous array. Mater. Sci. Semicond. Process..

[42] Liu J., Yuan Q., Huang W., Song X. (2021). A novel nanoporous Mg-Li material for efficient hydrogen generation. J. Magnes. Alloy..

[43] Srimurugan V., Jothiprakash C.G., Souparnika V., Prasanth R. (2022). Photocorrosion-less stable heterojunction photoanode for efficient visible-light driven solar hydrogen generation. Int. J. Hydrogen Energy.

[44] Ivanenko I., Ruda A., Povazhnyi V. (2022). Cobalt-nitrogen-doped activated carbons for hydrogen generation. Mater. Today Proc..

[45] Ali I., Imanova G.T., Mbianda X.Y., Alharbi O.M.L. (2022). Role of the radiations in water splitting for hydrogen generation. Sustain. Energy Technol. Assess..

[46] Ouyang L., Liu M., Chen K., Liu J., Wang H., Zhu M., Yartys V. (2022). Recent progress on hydrogen generation from the hydrolysis of light metals and hydrides. J. Alloy. Compd..

[47] Hadia N.M.A., Abdelazeez A.A.A., Alzaid M., Shaban M., Mohamed S.H., Hoex B., Hajjiah A., Rabia M. (2022). Converting Sewage Water into H2 Fuel Gas Using Cu/CuO Nanoporous Photocatalytic Electrodes. Materials.

[48] Rabia M., Mohamed S.H., Zhao H., Shaban M., Lei Y., Ahmed A.M. (2020). TiO_2_/TiO_x_N_Y_ hollow mushrooms-like nanocomposite photoanode for hydrogen electrogeneration. J. Porous Mater..

[49] Ahmed A.M., Rabia M., Shaban M. (2020). The structure and photoelectrochemical activity of Cr-doped PbS thin films grown by chemical bath deposition. RSC Adv..

[50] Rabia M., Shaban M., Adel A., Abdel-Khaliek A.A. (2019). Effect of plasmonic au nanoparticles on the photoactivity of polyaniline/indium tin oxide electrodes for water splitting. Environ. Prog. Sustain. Energy.

[51] Shaban M., Rabia M., Eldakrory M.G., Maree R.M., Ahmed A.M. (2020). Efficient photoselectrochemical hydrogen production utilizing of APbI_3_ (A = Na, Cs, and Li) perovskites nanorods. Int. J. Energy Res..

[52] Ragupathi V., Raja M.A., Panigrahi P., Ganapathi Subramaniam N. (2020). CuO/g-C_3_N_4_ nanocomposite as promising photocatalyst for photoelectrochemical water splitting. Optik.

[53] Huang X., Zhang M., Sun R., Long G., Liu Y., Zhao W. (2019). Enhanced hydrogen evolution from CuOx-C/TiO_2_ with multiple electron transport pathways. PLoS ONE.

[54] Naldoni A., Guler U., Wang Z., Marelli M., Malara F., Meng X., Besteiro L.V., Govorov A.O., Kildishev A.V., Boltasseva A. (2017). Broadband Hot-Electron Collection for Solar Water Splitting with Plasmonic Titanium Nitride. Adv. Opt. Mater..

[55] Abdelazeez A.A.A., Hadia N.M.A., Alzaid M., Shaban M., Mourad A.H.I., Fernández S., Rabia M. (2022). Development of CuO nanoporous material as a highly efficient optoelectronic device. Appl. Phys. A Mater. Sci. Process..

[56] Becke A.D. (1998). A new mixing of Hartree–Fock and local density-functional theories. J. Chem. Phys..

[57] Tönsing C., Timmer J., Kreutz C. (2019). Optimal Paths Between Parameter Estimates in Non-linear ODE Systems Using the Nudged Elastic Band Method. Front. Phys..

