# Supplementary material for: Conversion of Sewage Water into H2 Gas Fuel Using Hexagonal Nanosheets of the Polyaniline-Assisted Deposition of PbI2 as a Nanocomposite Photocathode with the Theoretical Qualitative Ab-Initio Calculation of the H2O Splitting
Source: Polymers (Basel). 2022 May 25;14(11):2148. doi: 10.3390/polym14112148 (PMC9183036; doi:10.3390/polym14112148)
Supplement: Supplementary file 1 [file polymers-14-02148-s001.zip › polymers-1640342-supplementary.pdf]

polymers MDPI

Supplementary file

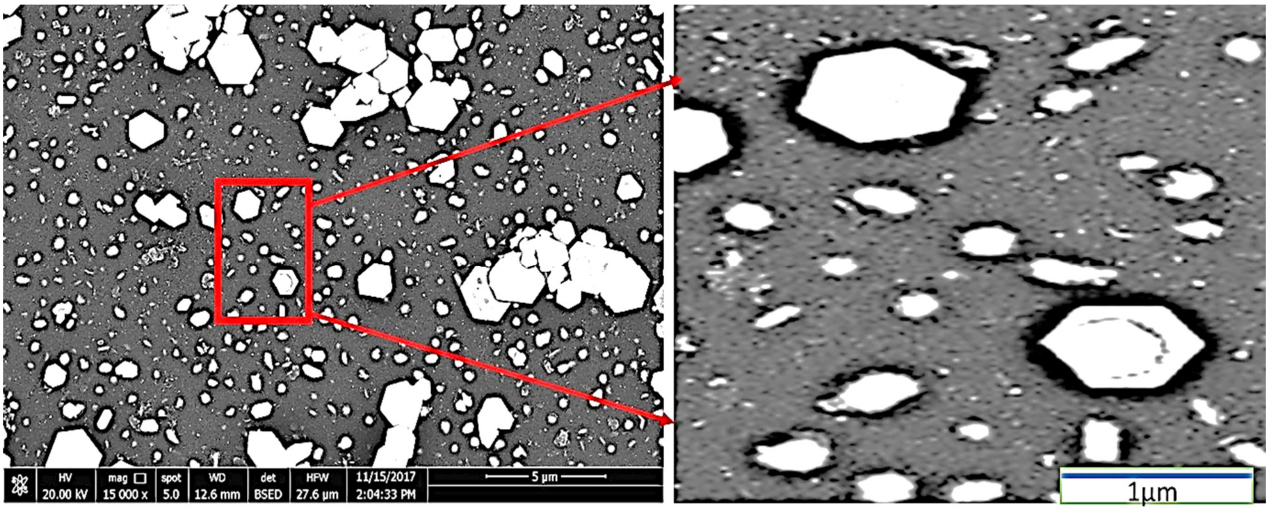

Figure S1. The SEM of PANI/PbI_2 under different scale bars (**a**) 5µm and (**b**) 1 µm.

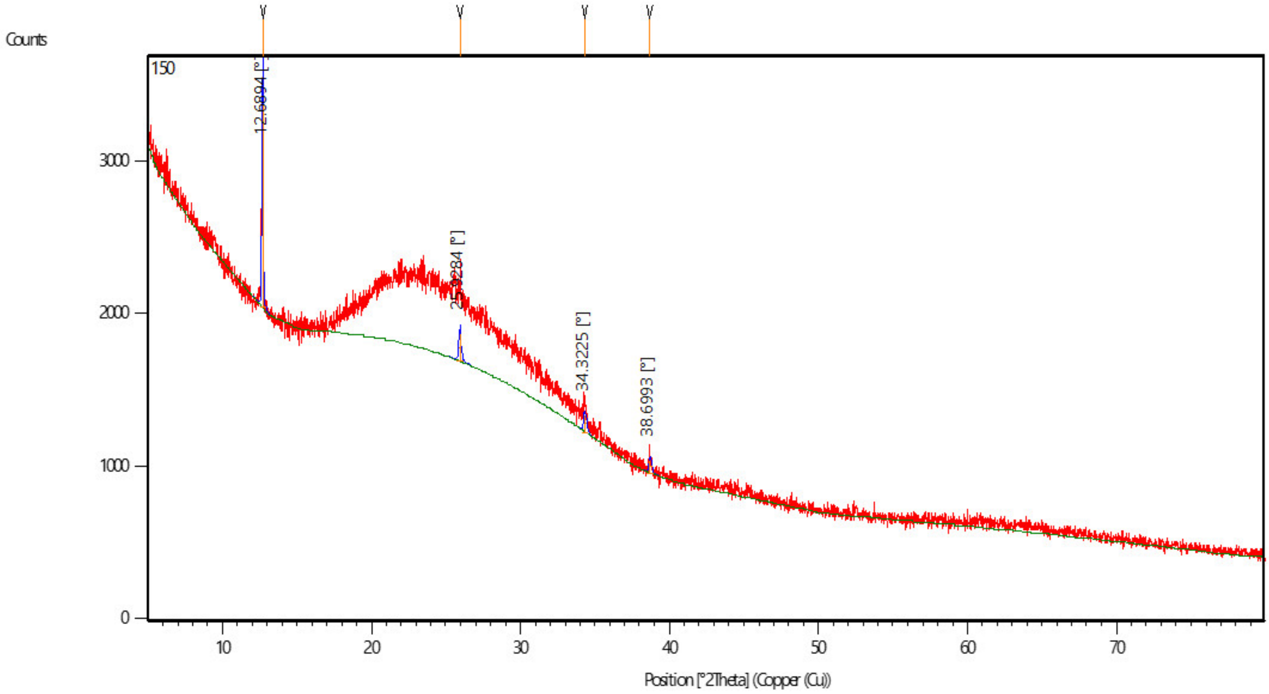

Figure S2. The standard XRD for PANI/PbI_2.
